# Are there different phenotypes of thoracic surgery patients? A latent class analysis of pretreatment patient-reported outcomes

**DOI:** 10.1016/j.xjon.2025.10.015

**Published:** 2025-10-27

**Authors:** Eagan J. Peters, Brenden Dufault, Sadeesh K. Srinathan, Gordon Buduhan, Lawrence Tan, Biniam Kidane

**Affiliations:** a Department of Medicine, Temerty Faculty of Medicine, University of Toronto, Toronto, Ontario, Canada; b Department of Community Health Sciences, Rady Faculty of Health Sciences, University of Manitoba, Winnipeg, Manitoba, Canada; c George & Fay Yee Centre for Healthcare Innovation, University of Manitoba, Winnipeg, Manitoba, Canada; d Section of Thoracic Surgery, Department of Surgery, Rady Faculty of Health Sciences, University of Manitoba, Winnipeg, Manitoba, Canada; e Population Health Research Institute, McMaster University, Hamilton, Ontario, Canada; f Division of Thoracic Surgery, Faculty of Medicine, University of British Columbia, Kelowna, British Columbia, Canada; g CancerCare Manitoba Research Institute, University of Manitoba, Winnipeg, Manitoba, Canada; h Department of Physiology & Pathophysiology, University of Manitoba, Winnipeg, Manitoba, Canada; i Department of Biomedical Engineering, University of Manitoba, Winnipeg, Manitoba, Canada

**Keywords:** esophageal cancer, EuroQol-5-dimension 3-level, frailty, health-related quality-of-life, latent class analysis, lung cancer, perioperative pain

## Abstract

**Background:**

Among patients undergoing thoracic surgery, quality of life is associated with multiple perioperative outcomes. Whether patients suffer reduced quality of life in certain areas compared to others is unclear. Knowing this could direct risk mitigation interventions for patients who share common symptoms. The objective of this study was to determine whether patients can be subdivided into groups based on preoperative quality of life.

**Methods:**

This is a secondary analysis of a retrospective cohort study of consecutive patients undergoing thoracic surgery between January 2018 and January 2019 at a Canadian tertiary center. Latent class analysis was conducted according to 3-level EuroQol-5 dimension (EQ-5D-3L) scores. The number of latent classes was selected by comparing different models using the Akaike information criterion, Bayesian information criterion, and *G*^2^ statistic. Class separation was measured using normalized entropy statistics.

**Results:**

Among 482 patients, models with 2 to 5 classes were constructed. The 3-class model demonstrated the lowest Akaike and Bayesian information criterion values. The *G*^2^ statistic and entropy showed increased preference for models as the number of classes decreased. Within the 3-class model, class 1 demonstrated a 73% to 100% probability of endorsing low impairment across all EQ-5D-3L dimensions, class 2 demonstrated a 93% probability of at least some impairment in mobility, and class 3 showed an 81% probability of moderate pain.

**Conclusions:**

There is evidence that patients undergoing thoracic surgery can be divided into 3 latent classes based on EQ-5D-3L score: low symptom burden, mobility-pain complex, and pain predominant. By identifying patients using these latent classes, targeted supportive interventions may be offered in the pretreatment period to improve perioperative outcomes.

## Graphical Abstract

**Figure:**
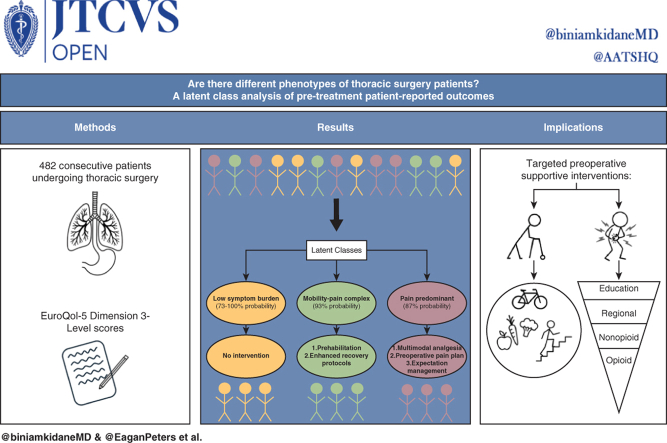

**Figure:**
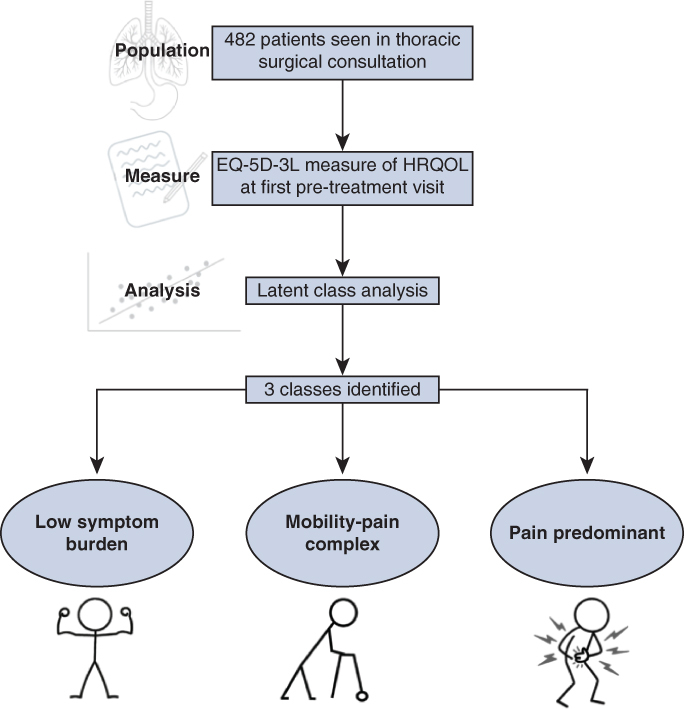
Latent classes of thoracic surgery patients based on health-related quality of life.

**Central Message**

Patients undergoing thoracic surgery exhibit 3 pretreatment phenotypes: low symptom burden, mobility-pain complex, and pain predominant. Targeted interventions may improve perioperative outcomes.

**Perspective**

Among patients undergoing thoracic surgery, pretreatment quality of life may be impaired in systematically different ways. Latent class analysis using the EQ-5D-3L survey identifies 3 patient phenotypes present prior to treatment: low symptom burden, mobility-pain complex, and pain predominant. Supportive interventions targeted at these phenotypes may improve perioperative outcomes.

Lung and esophageal cancer are the first- and seventh-leading causes of cancer-related death worldwide.^1^ Thoracic surgery is a primary modality for curative or palliative treatment.^2^^,^^3^ There is growing evidence that quality of life measures can inform the perioperative trajectory of patients undergoing thoracic surgery.^4^ Lung and esophageal cancers negatively affect patients’ quality of life,^5^^,^^6^ which may worsen further after surgery.^7^^,^^8^ Higher baseline quality of life prior to thoracic surgery is associated with improved postoperative survival.^9^^,^^10^ Among patients with lung cancer, lower preoperative quality of life is associated with postoperative complications and increased length of stay.^11^^,^^12^ For esophageal cancers, quality of life measures can distinguish between clinical T stages and are a superior prognosticator of survival compared to clinician-assigned measures, such as performance status.^13^^,^^14^

We previously demonstrated that overall preoperative health-related quality of life (HRQOL) is associated with the incidence of postoperative complications in patients undergoing a variety of thoracic surgeries for benign and malignant conditions.^15^ A knowledge gaps exists as to whether some patients suffer reduced HRQOL in systematically different ways compared to others, even at baseline prior to treatment. Knowing this, and being able to identify it pretreatment, could help direct targeted preoperative risk mitigation interventions for groups who share common symptom burdens or “phenotypes.” Thus, the objective of this study was to determine whether patients undergoing thoracic surgery could be subdivided into discrete cohesive groups, or “phenotypes,” based on pretreatment HRQOL measurements.

## Methods

### Study Population

This is a secondary analysis of a retrospective cohort of prospectively collected data. Institutional review board approval was obtained via the University of Manitoba Health Research Ethics Board (approval HS22804, approved April 25, 2019). Informed consent was waived. Consecutive adult patients undergoing elective thoracic surgery between January 2018 and January 2019 at a single Canadian tertiary hospital were included. Patients undergoing emergent surgery were excluded. Patient comorbidities were evaluated prospectively with the weighted Charlson Comorbidity Index.

### Indicator Variable: HRQOL Measure

The primary indicator variable was HRQOL, as self-reported by patients on the 3-level EuroQol-5-dimension (EQ-5D-3L) survey. These surveys were completed by patients at their first consultation visit, prior to delivery of any treatments, and prior to the actual consultation itself. The EQ-5D-3L is a validated generic measure of HRQOL that contains a descriptive scale of 5 HRQOL dimensions: mobility, anxiety and depression, pain and discomfort, usual activities, and self-care.^16^ These are scored as 1, 2, or 3, which correspond to mild, moderate, or severe impairment/burden of the HRQOL dimension.

### Outcomes

Because this was an exploratory study to determine whether distinct latent classes of patients exist based on the indicator variable, there were no primary or secondary outcomes of interest.

### Statistical Analysis

A latent class analysis was performed to identify clusters from the discrete responses to EQ-5D-3L items. Latent class analysis is a person-oriented clustering technique that can help elucidate subgroups or phenotypes as defined by their response probabilities across a potentially large number of observed variables and response patterns.^17^ Conditional probabilities distinguish the latent classes and may offer clinically useful descriptions of their symptom burden. The number of clusters was set at 2 to 5 in advance. The expectation-maximization algorithm was used to estimate both the item response probabilities for each group and the relative group sizes, expressed as percentage. This was performed multiple times, each time varying the number of classes and then selecting the best-fitting model. Relative model fit was measured using the Akaike information criterion and Bayesian information criterion, which penalize fit by accounting for the model's complexity. Absolute fit was measured using the *G*^2^ statistic, a likelihood ratio deviance measure assessing absolute fit. Separation between latent classes, which quantifies how distinct the response probabilities are between classes and how homogeneous they are within each class, was measured using the entropy statistic, with higher values denoting better class separation. When entropy is high, most patients can be assigned to a single latent class with high probability based on their observed responses. Patients with missing EQ-5D-3L data were omitted from the latent class analysis.

Each model was estimated with 10 random starts to avoid locally optimal solutions and thus obtain better fitting models. In addition to model fit statistics, clinical judgment and parsimony were used to help select the number of latent classes. The analysis was performed with R using the poLCA package, version 1.4.1. Sensitivity analysis was performed using 2 methods. First, full-information maximum likelihood was used, in which subjects with partial missing EQ-5D-3L data still contribute to the model estimation. This yields valid results if the missing data are missing at random. Full-information maximum likelihood was estimated by setting (na.rm = FALSE) in the poLCA function. Second, extreme values were imputed for all observation at both the minimum and maximum EQ-5D-3L values and compared.

## Results

A total of 515 patients were enrolled and analyzed. After exclusion of patients with missing EQ-5D-3L data, the final sample comprised 482 patients. Baseline patient demographic, clinical, and surgical characteristics are shown in Table 1.

**Table 1:** Demographic, clinical, and surgical characteristics of the study population (N = 515)

Variable	Value
Female sex, n (%)	242 (47.0)
Age, y, median (range)	66.3 (16.3-91.9)
BMI, mean (SD)	28.9 (5.9)
Pack-years, median (range)	13.0 (0.0-130.0)
Married, n (%)	337 (65.7)
FEV1, %, mean (SD)	86.3 (20.7)
WCCI, median (range)	3.0 (0.0-11.0)
Pulmonary disease, n (%)	106 (20.6)
Nonmetastatic cancer, n (%)	272 (52.8)
Metastatic cancer, n (%)	86 (16.7)
Type of operation, n (%)	
Sublobar resection	154 (29.9)
Lobectomy, bilobectomy, or pneumonectomy	131 (25.4)
Other pleural or endoscopic operation	107 (20.8)
Benign esophageal or diaphragmatic hernia surgery	48 (9.3)
Mediastinal mass resection	31 (6.0)
Esophagectomy or total gastrectomy	24 (4.7)
Chest wall and extended resection	20 (3.9)
Minimally invasive surgery, n (%)	423 (82.1)
Cancer, n (%)	345 (67.0)
Adenocarcinoma, n (%)	194 (37.7)
Stage, n (%)	
0	3 (0.9)
1	133 (40.8)
2	50 (9.7)
3	47 (9.1)
4	93 (18.1)
Non–small cell lung cancer, n (%)	208 (40.4)

*BMI*, Body mass index; *SD*, standard deviation; *FEV1*, forced expiratory volume in 1 second as a percent of the average of population-matched sample; *WCCI*, weighted Charlson Comorbidity Index.

The full latent class analysis is provided in the Appendix E1 as Table E1, Table E2, Table E3, Table E4. The 5 latent class analysis models’ fit to the data is compared in Table 2. The 3-class model demonstrated the lowest Akaike information criterion and Bayesian information criterion values, implying best relative fit compared to other models. The *G*^2^ statistic showed increasing preference for models as the number of classes increased. This was expected, given that more complex models will always lead to improved fit. There was a sharp reduction in the *G*^2^ statistic value (and thus improvement) up to the 3-class solution, after which the change in value slowed, reflecting diminishing returns of complexity. The entropy statistic was large for all models and less useful for comparison here. Statistically, the best-fitting model was deemed to be the 3-class solution. This is because the Akaike information criterion and Bayesian information criterion were superior for the 3-class solution and the entropy values were almost identical across all classes. In addition, classes with superior *G*^2^ statistic values were subverted by disproportionately small class sizes compared to others. These include class 1 in the 4-class solution and classes 1 and 2 in the 5-class solution (Appendix E1). Because these class sizes were as small as 4.5% to 4.8% of patients, their clinical utility was reduced.The result of the three-class analysis, the best-fitting model, are provided in Table 3. Class 1 demonstrated a probability to endorse low impairment across all EQ-5D-3L dimensions ranging from 73.16% to 100%. Class 2 demonstrated a 91.07% probability of at least some impairment in mobility, a 55.14% probability of a moderate pain/discomfort burden, and a 28.64% probability of a high pain/discomfort burden. Class 3 showed an 80.96% probability of moderate pain/discomfort. Sensitivity analysis showed that full-information maximum likelihood still preferred the 3-class solution. The 3-class parameters were similar to those reported in Table 3; therefore, interpretation did not change under the first sensitivity method. When imputing a value of 1 (best level) for all missing EQ-5D-3L items, the latent class analysis results also were similar. The analysis results changed only when the assumption was made that every missing EQ-5D-3L response was 3 (ie, the worst level). In this case, the Akaike information criterion and the Bayesian information criterion statistics preferred a 4-class solution. When this occurred, the first 3 classes were qualitatively the same as those reported in Table 3, and the fourth class was simply those with a response of 3 for all items with a probability of 1.0000. This is tautologically true and almost certainly unlikely the case.

**Table 2:** Comparison of the 5 latent class analysis models according to model fit statistics

Model	AIC	BIC	*G*^2^ statistic	Entropy
1 class	3506	3548	595.93	1.0000
2 class	3096	3184	164.09	0.9954
3 class	3041	3175	87.52	0.9927
4 class	3049	3229	73.31	0.9908
5 class	3062	3288	64.64	0.9893

*AIC*, Akaike information criterion; *BIC*, Bayesian information criterion.

**Table 3:** Latent class analysis model with 3 classes

Model	Probability, low impairment	Probability, some impairment	Probability, high impairment
EQ-5D-3L mobility			
Class 1	0.9741	0.0259	0.0000
Class 2	0.0653	0.9107	0.0239
Class 3	0.4768	0.5232	0.0000
EQ-5D-3L self-care			
Class 1	1.0000	0.0000	0.0000
Class 2	0.3175	0.5987	0.0838
Class 3	1.0000	0.0000	0.0300
EQ-5D-3L usual activities			
Class 1	0.9646	0.0354	0.0000
Class 2	0.0387	0.6878	0.2735
Class 3	0.2815	0.6885	0.0300
EQ-5D-3L pain/discomfort			
Class 1	0.7316	0.2676	0.0009
Class 2	0.1622	0.5514	0.2864
Class 3	0.1446	0.8096	0.0458
EQ-5D-3L anxiety/depression			
Class 1	0.7841	0.1812	0.0348
Class 2	0.3215	0.5332	0.1453
Class 3	0.5849	0.4151	0.0000

*EQ-5D-3L*, EuroQol-5-dimension 3-level.

## Discussion

**Figure 1:**
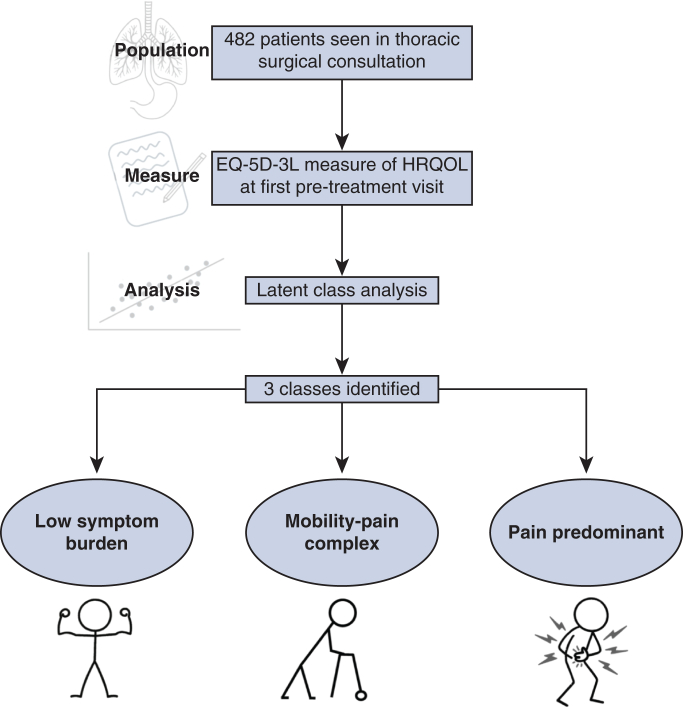
Flow diagram summarizing the population, measure, analysis, and clinically relevant phenotypes identified by the latent class analysis.

Our results demonstrate that patients referred to thoracic surgical practice for a variety of conditions (but predominantly malignant) can be grouped into latent clusters or phenotypes based on EQ-5D-3L scores. The 3-class model appears to have the best relative fit compared to others. These three classes of patients can be divided into clinically meaningful clusters, as depicted in Figure 1 and are described herein:

### Class 1: Low Symptom Burden

The probability of the low symptom burden cluster of patients to endorse impairments in mobility, social, or usual activities was low (0-3.54%). Only 21.59% of patients endorsed any problems with anxiety/depression, and 26.84% endorsed any problems with pain/discomfort. If identified, these patients likely would not benefit from targeted supportive interventions to improve aspects of HRQOL. It is possible that these patients are younger, have earlier-stage disease, or have a lower comorbidity burden. In the context of previous literature, quality of life among patients undergoing thoracic surgery shows age-related perioperative stratification.^18^ Patients with early-stage lung cancer are known to have higher HRQOL than those with advanced-stage disease.^19^ Patients undergoing thoracic surgery for lung cancer who smoke have worse perioperative quality of life,^20^ as do patients undergoing surgery both for both lung and esophageal cancer who have an increased medical comorbidity burden.^21^^,^^22^ Nonetheless, these findings should be validated in an external patient population.

### Class 2: Mobility-Pain Complex

The probability of this class of patients to endorse mobility impairment was 91.07% and 2.39% for moderate and severe impairments, respectively. There was also a dual pain impairment in this group, with a 55.14% and 28.64% probability of moderate and severe pain burden, respectively. The characteristics of this class are likely multifactorial. First, our data demonstrate that patients presenting for thoracic surgery are more likely to be of advanced age, in keeping with previous reports.^23^ Among older patients undergoing early-stage lung cancer resection, reduction in perioperative mobility has been associated with changes in HRQOL.^24^ Second, it has been shown that preoperative forced expiratory volume in 1 second and CO diffusion capacity of the lungs are associated with perioperative HRQOL among patients undergoing lung cancer resection.^25^^,^^26^ Third, it has been established in multiple cohorts that patients with lung and esophageal cancer who are designated as frail have a greater likelihood of worse perioperative HRQOL.^27^^,^^28^ Therefore, there is likely significant overlap between patients who score high on mobility impairment and those who are older, have reduced pulmonary function, and are frail.

Frailty is likely the most parsimonious measure of what these multiple factors are approximating. As such, mobility-impaired patients could be strong candidates for targeted supportive interventions, such as “prehabilitation” and enhanced recovery after surgery protocols aimed at reducing the frailty burden on patients in the perioperative period.^29^^,^^30^ Examples of these evidence-based interventions include supervised cycling and walking training, home stair climbing and transfer exercises, diaphragmatic breathing and airway clearance therapy, anemia correction, nutrition optimization, early mobilization, and multimodal analgesia, among others. A major benefit of using the EQ-5D-3L survey as a screen to identify this “frail” phenotype is that it is a much simpler and easier test/questionnaire than any validated frailty questionnaires/instruments. Compared to existing frailty instruments, the EQ-5D-3L has shown convergent and discriminant validity.^31^^,^^32^ As such, in the ambulatory thoracic surgery setting, where frailty measurements are not routine, the EQ-5D-3L can serve as a rapid screening measure to determine the presence of frailty, among other elements of HRQOL. If identified, additional frailty measurements then can be performed on a case-by-case basis if it will help inform management.

### Class 3: Pain Predominant

The pain predominant cluster of patients had an 80.96% probability of endorsing moderate pain and 4.58% probability of endorsing severe pain on pretreatment assessment. It is possible that the pain experienced by these patients contributed to the small subset who experienced high impairment probabilities in self-care and usual activities as well. This cluster is consistent with previous literature describing a subset of patients who experience preoperative thoracic pain and are correspondingly at increased likelihood of postoperative pain.^33^ Options for pain-specific interventions in the perioperative period include multimodal oral analgesia,^34^ regional anesthesia using liposomal or bupivacaine-based intercostal nerve blocks,^35^^,^^36^ thoracic epidural and paravertebral blockade,^37^ preemptive activation of an acute/transitional pain service team,^38^ and expectation management strategies.^39^ If patients belonging to the pain predominant class are to undergo preoperative anesthesia consultation for medical optimization, they also could be flagged concurrently for high risk of perioperative pain, akin to existing transitional pain service protocols. In doing so, an analgesic plan for the patient may be identified in advance according to the aforementioned protocols, such as preoperative opioid reduction and opioid-sparing additions, perioperative interdisciplinary involvement with physiotherapy and clinical psychology, and postoperative outpatient pain clinic follow-up to supervise return to pain and function back to pretreatment levels.^38^ These analgesic plans also may be further personalized to patients’ unique needs (eg, addition of ketamine for those with chronic pain and/or preexisting long-term opioid use, glucocorticoids for those who may also wish to prevent postoperative nausea/vomiting).^30^

### Comparison With Previous Reports

To our knowledge, there is no previously published latent class analysis on a variety of thoracic surgery patients in the preoperative period based on simple routine HRQOL measure. However, a previous latent class analysis according to preoperative physical dysfunction for elderly patients with early-stage lung cancer also identified 3 classes: “anxiety/depression emotion-poor sleep,” “frailty of physical function,” and “pulmonary hypofunction-low activity tolerance.”^40^ Our results likely encompass these latter 2 groups by virtue of our mobility-pain complex class. Furthermore, we did not identify a class based on anxiety/depression. These differences likely are related in large part to the difference in indicator variable used (ie, physical dysfunction vs HRQOL). Although there is a published latent class analysis based on EQ-5D, this was conducted among patients exclusively with lung cancer at an average of 4.8 months and >1 year postdiagnosis as opposed to the pretreatment period.^E1^ The authors identified 4 classes: “pain dominant impairment,” “mobility/usual activities impairment,” “good HRQOL,” and “poor HRQOL.” The first 3 classes are in line with our 3 classes; however, because the HRQOL measurements occurred at different timepoints in the treatment pathway (pretreatment in our study vs 4.8 months to >1 year postdiagnosis in the study by Zhang and colleagues^40^), we cannot draw meaningful conclusions about how they relate to one another. They are assessing fundamentally different constructs. Applying HRQOL measures to identify at-risk phenotypes prior to treatment is important to developing preventive treatment strategies.

### Limitations

This study is limited by its single-center nature. To improve external validity, replication with external populations is important in latent class analysis. Multicenter validation will confirm phenotype stability across diverse populations. This is because latent class analysis assigns patients to classes based on their probability of being in a class given their pattern of scores on an indicator variable. However, we do not know whether other cohorts of patients undergoing thoracic surgery would fit our classes based on the same indicator variable we used (ie, EQ-5D-3L). For example, an argument can be made that we could have classified our patient population along usual activities in class 1 or anxiety/depression in class 2. Additional independent studies that repeat our analysis using the EQ-5D-3L will help either affirm or refine our classification in terms of both the quantity and the characteristics of each class identified.

Investigators may be hesitant to implement the EQ-5D-3L in busy preoperative clinics given the already substantial documentation burden on providers and survey burden on patients. Fortunately, this may be offset by the fact that the survey is short (limited to 5 descriptive Likert scales and 1 visual analog scale) and cognitively undemanding and takes only a few minutes to complete.^E2^ It also has proven feasibility in millions of patients.^E3^ The present study offers an example of the EQ-5D-3L's feasibility in a busy thoracic surgery clinic that was sustained for longer than 1 year without interruption to usual clinic flow or patient care. However, it may be prudent for future studies to incorporate the 5-level as opposed to the 3-level EQ-5D survey, as its superior sensitivity may account for subtle variations in HRQOL and ameliorate any potential ceiling effect.^E4^

By virtue of this study's retrospective design, there is a risk of reporting bias. In addition, selection bias may have been introduced by the 33 patients who had missing data and thus were not included in the analysis. If these data are not missing at random, then our results may be subverted. We also must emphasize that while clustering patients may offer useful clinical insights into broad patterns, each patient will be unique and deviate to some extent from their assigned latent class.

### Strengths

Given the study's large sample size, the number of patients was likely adequate for detecting the “true” number of latent classes based on previous studies estimating sample sizes required to perform with high accuracy in latent class analysis.^17^ We also used multiple fit indices to help determine the optimal number of classes to select. With regard to measurement of the indicator variable itself, the EQ-5D-3L is a standardized and well-validated instrument for patients with a variety of benign and malignant thoracic diseases.^16^^,^^E5^ Moreover, the data were collected prospectively among consecutive patients, thus helping reduce selection bias.

## Conclusions

This study demonstrates that patients referred to thoracic surgical practice for a variety of (but predominantly malignant) conditions can be grouped into latent clusters or “phenotypes” based on EQ-5D-3L scores: low symptom burden, mobility-pain complex, and pain predominant. By identifying patients using these latent classes, targeted supportive interventions may be offered in the pretreatment period to improve perioperative outcomes. Future studies can assess whether targeted interventions using the latent clusters can improve (or at least attenuate impact of) treatment-related HRQOL changes. Subgroup analysis of latent classes according to covariates such as baseline demographics and cancer status is also required. While we have previously demonstrated that preoperative EQ-5D-3L visual analog scale measures are independently associated with the development of postoperative complications, it also should be determined whether the latent classes as determined by EQ-5D-3L are associated with postoperative complications. Future research is needed to determine associations between these phenotypes and such postoperative outcomes. Ultimately, the latent classes identified will require replicability among other cohorts to confirm their validity. Thus, our findings can provide a foundation for future studies seeking to apply latent class grouping to patients undergoing thoracic surgery.

## Conflict of Interest Statement

The authors reported no conflicts of interest.

The *Journal* policy requires editors and reviewers to disclose conflicts of interest and to decline handling or reviewing manuscripts for which they may have a conflict of interest. The editors and reviewers of this article have no conflicts of interest.

## Figures and Tables

**Table E1:** Latent class analysis model with 2 classes

Model	Probability, low impairment	Probability, some impairment	Probability, high impairment
EQ-5D-3L mobility			
Class 1	0.9140	0.0860	0.0000
Class 2	0.2236	0.7660	0.0104
EQ-5D-3L self-care			
Class 1	1.0000	0.0000	0.0000
Class 2	0.7031	0.2604	0.0365
EQ-5D-3L usual activities			
Class 1	0.8779	0.1221	0.0000
Class 2	0.0801	0.7741	0.1459
EQ-5D-3L pain/discomfort			
Class 1	0.6197	0.3773	0.0030
Class 2	0.1265	0.7114	0.1621
EQ-5D-3L anxiety/depression			
Class 1	0.7671	0.2068	0.0261
Class 2	0.4299	0.5054	0.0647

*EQ-5D-3L*, EuroQol-5-dimension 3-level.

**Table E2:** Latent class analysis model with 3 classes

Model	Probability, low impairment	Probability, some impairment	Probability, high impairment
EQ-5D-3L mobility			
Class 1	0.9741	0.0259	0.0000
Class 2	0.0653	0.9107	0.0239
Class 3	0.4768	0.5232	0.0000
EQ-5D-3L self-care			
Class 1	1.0000	0.0000	0.0000
Class 2	0.3175	0.5987	0.2735
Class 3	1.0000	0.0000	0.0300
EQ-5D-3L usual activities			
Class 1	0.9646	0.0354	0.0000
Class 2	0.0387	0.6878	0.2735
Class 3	0.2815	0.6885	0.0300
EQ-5D-3L pain/discomfort			
Class 1	0.7316	0.2676	0.0.009
Class 2	0.1622	0.5514	0.2864
Class 3	0.1446	0.8096	0.0458
EQ-5D-3L anxiety/depression			
Class 1	0.7841	0.1812	0.0348
Class 2	0.3215	0.5332	0.1453
Class 3	0.5849	0.4151	0.0000

*EQ-5D-3L*, EuroQol-5-dimension 3-level.

**Table E3:** Latent class analysis model with 4 classes

Model	Probability, low impairment	Probability, some impairment	Probability, high impairment
EQ-5D-3L mobility			
Class 1	0.0000	0.9117	0.0883
Class 2	0.4733	0.5267	0.0000
Class 3	0.0874	0.9126	0.0000
Class 4	0.9709	0.0291	0.0000
EQ-5D-3L self-care			
Class 1	0.4574	0.3469	0.1957
Class 2	1.0000	0.0000	0.0000
Class 3	0.2583	0.6991	0.0426
Class 4	1.0000	0.0000	0.0000
EQ-5D-3L usual activities			
Class 1	0.1899	0.5158	0.2943
Class 2	0.2635	0.7041	0.0324
Class 3	0.0000	0.7376	0.2624
Class 4	0.9651	0.0349	0.0000
EQ-5D-3L pain/discomfort			
Class 1	0.0000	0.3274	0.6726
Class 2	0.1449	0.8167	0.0384
Class 3	0.2174	0.6195	0.1631
Class 4	0.7264	0.2719	0.0017
EQ-5D-3L anxiety/depression			
Class 1	0.0000	0.7316	0.2684
Class 2	0.5887	0.4113	0.0000
Class 3	0.4266	0.4736	0.0998
Class 4	0.7825	0.1829	0.0345

*EQ-5D-3L*, EuroQol-5-dimension 3-level.

**Table E4:** Latent class analysis model with 5 classes

Model	Probability, low impairment	Probability, some impairment	Probability, high impairment
EQ-5D-3L mobility			
Class 1	0.0000	0.9142	0.0858
Class 2	0.0000	1.0000	0.0000
Class 3	0.1236	0.8764	0.0000
Class 4	0.4658	0.5342	0.0000
Class 5	0.9653	0.0347	0.0000
EQ-5D-3L self-care			
Class 1	0.4605	0.3486	0.1909
Class 2	0.4248	0.5752	0.0000
Class 3	0.1422	0.7892	0.0686
Class 4	1.0000	0.0000	0.0000
Class 5	1.0000	0.0000	0.0000
EQ-5D-3L usual activities			
Class 1	0.1730	0.5516	0.2754
Class 2	0.0000	0.8936	0.1064
Class 3	0.0116	0.6358	0.3526
Class 4	0.2510	0.7118	0.0372
Class 5	0.9551	0.0449	0.0000
EQ-5D-3L pain/discomfort			
Class 1	0.0000	0.2527	0.7473
Class 2	0.5892	0.0000	0.4108
Class 3	0.0763	0.9237	0.0000
Class 4	0.1109	0.8583	0.0308
Class 5	0.7247	0.2728	0.0025
EQ-5D-3L anxiety/depression			
Class 1	0.0000	0.7079	0.2921
Class 2	0.5975	0.4025	0.0000
Class 3	0.3426	0.5175	0.1399
Class 4	0.5834	0.4166	0.0000
Class 5	0.7797	0.1863	0.0340

*EQ-5D-3L*, EuroQol-5-dimension 3-level.

## Appendix E1 Outputs

### LCA Model With 2 Classes

## Conditional item response (column) probabilities,

## by outcome variable, for each class (row)

##

## $EQ5D_M1

## Pr(1) Pr(2) Pr(3)

## class 1: 0.9140 0.086 0.0000

## class 2: 0.2236 0.766 0.0104

##

## $EQ5D_S1

## Pr(1) Pr(2) Pr(3)

## class 1: 1.0000 0.0000 0.0000

## class 2: 0.7031 0.2604 0.0365

##

## $EQ5D_U1

## Pr(1) Pr(2) Pr(3)

## class 1: 0.8779 0.1221 0.0000

## class 2: 0.0801 0.7741 0.1459

##

## $EQ5D_P1

## Pr(1) Pr(2) Pr(3)

## class 1: 0.6197 0.3773 0.0030

## class 2: 0.1265 0.7114 0.1621

##

## $EQ5D_A1

## Pr(1) Pr(2) Pr(3)

## class 1: 0.7671 0.2068 0.0261

## class 2: 0.4299 0.5054 0.0647

##

## Estimated class population shares

## 0.6017 0.3983

##

## Predicted class memberships (by modal posterior prob.)

## 0.6245 0.3755

##

## =========================================================

## Fit for 2 latent classes:

## =========================================================

## number of observations: 482

## number of estimated parameters: 21

## residual degrees of freedom: 221

## maximum log-likelihood: -1527

##

## AIC(2): 3096

## BIC(2): 3184

## Gˆ2(2): 164.1 (Likelihood ratio/deviance statistic)

## Xˆ2(2): 1354 (Chi-square goodness of fit)

##

### LCA Model With 3 Classes

## Conditional item response (column) probabilities,

## by outcome variable, for each class (row)

##

## $EQ5D_M1

## Pr(1) Pr(2) Pr(3)

## class 1: 0.9741 0.0259 0.0000

## class 2: 0.0653 0.9107 0.0239

## class 3: 0.4768 0.5232 0.0000

##

## $EQ5D_S1

## Pr(1) Pr(2) Pr(3)

## class 1: 1.0000 0.0000 0.0000

## class 2: 0.3175 0.5987 0.0838

## class 3: 1.0000 0.0000 0.0000

##

## $EQ5D_U1

## Pr(1) Pr(2) Pr(3)

## class 1: 0.9646 0.0354 0.0000

## class 2: 0.0387 0.6878 0.2735

## class 3: 0.2815 0.6885 0.0300

##

## $EQ5D_P1

## Pr(1) Pr(2) Pr(3)

## class 1: 0.7316 0.2676 0.0009

## class 2: 0.1622 0.5514 0.2864

## class 3: 0.1446 0.8096 0.0458

##

## $EQ5D_A1

## Pr(1) Pr(2) Pr(3)

## class 1: 0.7841 0.1812 0.0348

## class 2: 0.3215 0.5332 0.1453

## class 3: 0.5849 0.4151 0.0000

##

## Estimated class population shares

## 0.4695 0.1733 0.3572

##

## Predicted class memberships (by modal posterior prob.)

## 0.4979 0.1452 0.3568

##

## =========================================================

## Fit for 3 latent classes:

## =========================================================

## number of observations: 482

## number of estimated parameters: 32

## residual degrees of freedom: 210

## maximum log-likelihood: −1489

##

## AIC(3): 3041

## BIC(3): 3175

## Gˆ2(3): 87.52 (Likelihood ratio/deviance statistic)

## Xˆ2(3): 241.5 (Chi-square goodness of fit)

##

### LCA Model With 4 Classes

## Conditional item response (column) probabilities,

## by outcome variable, for each class (row)

##

## $EQ5D_M1

## Pr(1) Pr(2) Pr(3)

## class 1: 0.0000 0.9117 0.0883

## class 2: 0.4733 0.5267 0.0000

## class 3: 0.0874 0.9126 0.0000

## class 4: 0.9709 0.0291 0.0000

##

## $EQ5D_S1

## Pr(1) Pr(2) Pr(3)

## class 1: 0.4574 0.3469 0.1957

## class 2: 1.0000 0.0000 0.0000

## class 3: 0.2583 0.6991 0.0426

## class 4: 1.0000 0.0000 0.0000

##

## $EQ5D_U1

## Pr(1) Pr(2) Pr(3)

## class 1: 0.1899 0.5158 0.2943

## class 2: 0.2635 0.7041 0.0324

## class 3: 0.0000 0.7376 0.2624

## class 4: 0.9651 0.0349 0.0000

##

## $EQ5D_P1

## Pr(1) Pr(2) Pr(3)

## class 1: 0.0000 0.3274 0.6726

## class 2: 0.1449 0.8167 0.0384

## class 3: 0.2174 0.6195 0.1631

## class 4: 0.7264 0.2719 0.0017

##

## $EQ5D_A1

## Pr(1) Pr(2) Pr(3)

## class 1: 0.0000 0.7316 0.2684

## class 2: 0.5887 0.4113 0.0000

## class 3: 0.4266 0.4736 0.0998

## class 4: 0.7825 0.1829 0.0345

##

## Estimated class population shares

## 0.047 0.3532 0.1251 0.4747

##

## Predicted class memberships (by modal posterior prob.)

## 0.0456 0.3506 0.1058 0.4979

##

## =========================================================

## Fit for 4 latent classes:

## =========================================================

## number of observations: 482

## number of estimated parameters: 43

## residual degrees of freedom: 199

## maximum log-likelihood: −1482

##

## AIC(4): 3049

## BIC(4): 3229

## Gˆ2(4): 73.31 (Likelihood ratio/deviance statistic)

## Xˆ2(4): 96.44 (Chi-square goodness of fit)

##

### LCA Model With 5 Classes

## Conditional item response (column) probabilities,

## by outcome variable, for each class (row)

##

## $EQ5D_M1

## Pr(1) Pr(2) Pr(3)

## class 1: 0.0000 0.9142 0.0858

## class 2: 0.0000 1.0000 0.0000

## class 3: 0.1236 0.8764 0.0000

## class 4: 0.4658 0.5342 0.0000

## class 5: 0.9653 0.0347 0.0000

##

## $EQ5D_S1

## Pr(1) Pr(2) Pr(3)

## class 1: 0.4605 0.3486 0.1909

## class 2: 0.4248 0.5752 0.0000

## class 3: 0.1422 0.7892 0.0686

## class 4: 1.0000 0.0000 0.0000

## class 5: 1.0000 0.0000 0.0000

##

## $EQ5D_U1

## Pr(1) Pr(2) Pr(3)

## class 1: 0.1730 0.5516 0.2754

## class 2: 0.0000 0.8936 0.1064

## class 3: 0.0116 0.6358 0.3526

## class 4: 0.2510 0.7118 0.0372

## class 5: 0.9551 0.0449 0.0000

##

## $EQ5D_P1

## Pr(1) Pr(2) Pr(3)

## class 1: 0.0000 0.2527 0.7473

## class 2: 0.5892 0.0000 0.4108

## class 3: 0.0763 0.9237 0.0000

## class 4: 0.1109 0.8583 0.0308

## class 5: 0.7247 0.2728 0.0025

##

## $EQ5D_A1

## Pr(1) Pr(2) Pr(3)

## class 1: 0.0000 0.7079 0.2921

## class 2: 0.5975 0.4025 0.0000

## class 3: 0.3426 0.5175 0.1399

## class 4: 0.5834 0.4166 0.0000

## class 5: 0.7797 0.1863 0.0340

##

## Estimated class population shares

## 0.0483 0.0451 0.0772 0.3425 0.4868

##

## Predicted class memberships (by modal posterior prob.)

## 0.0519 0.0353 0.0685 0.3216 0.5228

##

## =========================================================

## Fit for 5 latent classes:

## =========================================================

## number of observations: 482

## number of estimated parameters: 54

## residual degrees of freedom: 188

## maximum log-likelihood: −1477

##

## AIC(5): 3062

## BIC(5): 3288

## Gˆ2(5): 64.64 (Likelihood ratio/deviance statistic)

19

## Xˆ2(5): 101.1 (Chi-square goodness of fit)

##
